# 8-Bromo-2-methyl­quinoline

**DOI:** 10.1107/S1600536809020625

**Published:** 2009-06-06

**Authors:** Lin-Tao Yang, Fang Shen, Jiao Ye, Tian-Quan Wu, Ai-Xi Hu

**Affiliations:** a College of Chemistry and Chemical Engineering, Hunan University, Changsha 410082, People’s Republic of China

## Abstract

In the crystal structure of the title compound, C_10_H_8_BrN, the dihedral angle between the two six-membered rings of the quinoline system is 0.49 (16)°. The mol­ecules are packed in a face-to-face arrangement fashion, with a centroid–centroid distance of 3.76 Å between the benzene and pyridine rings of adjacent mol­ecules. No hydrogen bonding is found in the crystal structure.

## Related literature

The title compound is an important inter­mediate in the pharmaceutical industry, see: Shen & Hartwig (2006 ▶); Ranu *et al.* (2000 ▶); Lee & Hartwig (2005 ▶). For related structures, see: Amini *et al.* (2008 ▶); Faza­eli *et al.* (2008 ▶); Sattarzadeh *et al.* (2009 ▶).
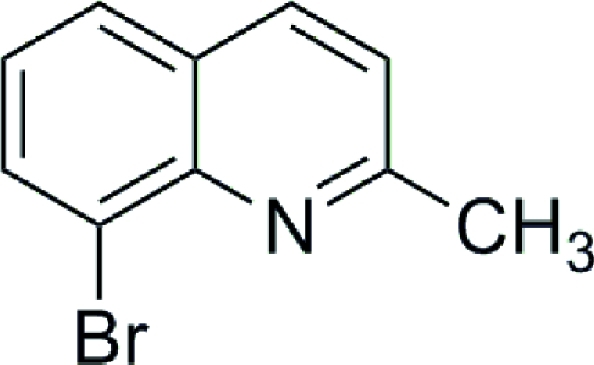


## Experimental

### 

#### Crystal data

C_10_H_8_BrN
*M* *_r_* = 222.08
Monoclinic, 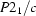

*a* = 5.0440 (17) Å
*b* = 13.467 (4) Å
*c* = 13.391 (4) Å
β = 97.678 (4)°
*V* = 901.4 (5) Å^3^
*Z* = 4
Mo *K*α radiation
μ = 4.50 mm^−1^
*T* = 291 K
0.36 × 0.31 × 0.28 mm

#### Data collection

Bruker SMART APEX CCD diffractometer
Absorption correction: multi-scan (*SADABS*; Sheldrick, 2004 ▶) *T*_min_ = 0.235, *T*_max_ = 0.286
4668 measured reflections
1765 independent reflections
1039 reflections with *I* > 2σ(*I*)
*R*_int_ = 0.156

#### Refinement

*R*[*F* ^2^ > 2σ(*F* ^2^)] = 0.071
*wR*(*F* ^2^) = 0.195
*S* = 1.01
1765 reflections
110 parameters
H-atom parameters constrained
Δρ_max_ = 0.88 e Å^−3^
Δρ_min_ = −0.91 e Å^−3^

### 

Data collection: *SMART* (Bruker, 2003 ▶); cell refinement: *SAINT-Plus* (Bruker, 2001 ▶); data reduction: *SAINT-Plus*; program(s) used to solve structure: *SHELXS97* (Sheldrick, 2008 ▶); program(s) used to refine structure: *SHELXL97* (Sheldrick, 2008 ▶); molecular graphics: *ORTEP-3 for Windows* (Farrugia, 1997 ▶); software used to prepare material for publication: *WinGX* (Farrugia, 1999 ▶).

## Supplementary Material

Crystal structure: contains datablocks I, New_Global_Publ_Block. DOI: 10.1107/S1600536809020625/xu2533sup1.cif

Structure factors: contains datablocks I. DOI: 10.1107/S1600536809020625/xu2533Isup2.hkl

Additional supplementary materials:  crystallographic information; 3D view; checkCIF report

## Figures and Tables

**Fig. 1.**
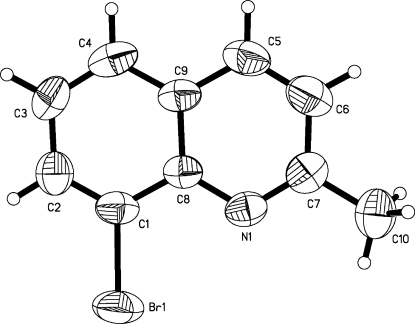
Molecular structure showing 30% probability displacement ellipsoids.

## supplementary crystallographic information

### Comment

The title compound, 8-bromo-2-methylquinoline, is an important intermediate of medcine industry (Shen & Hartwig, 2006; Ranu *et al.*, 2000; Lee & Hartwig, 2005). The unit-cell of the title compound contains four molecules, and the corresponding bond lengths and angles of these molecules are agree with each other. The molecules are stablizated by π-π stacking (centroids distance is 3.76 Å). Herein we report the synthesis and crystal structure of 8-bromo-2-methylquinoline. For more related structures, see: Amini *et al.*(2008), Fazaeli *et al.* (2008), Sattarzadeh *et al.* (2009).

### Experimental

A solution of 2-bromoaniline (0.05 mol), boric acid (3.10 g) and 18% HCl (50 ml) was heated to reflux. Then a mixture of crotonaldehyde (0.06 mol) and 2-bromonitrobenzene (0.01 mol) was slowly added with stirring in 1 h. The reaction mixture was subsequently stirred at 373 K for another 2.5 h, and then an equimolar amount of anhydrous ZnCl_2_ was added with vigorous stirring for 0.5 h. After the reaction was completed, the reaction solution was cooled in an ice bath and the crude brown solid was filtered, washed with 2-propanol, dissolved in the water, and neutralized with concentrated NH_3._H_2_O solution to pH of 8. After cool immersed, filtrated and air dried, the product was obtained as a grey solid. Yield: 52.0%. m.p. 342–343 K. Crystals suitable for X-ray structure determination were obtained by slow evaporation of an ethanol solution at room temperature.

### Refinement

The H-atoms were positioned geometrically, with C—H = 0.93 Å for aromatic, 0.96 Å for methyl, and refined as riding with *U*_iso_(H) = 1.2 or 1.5 *U*_eq_(C).

### Crystal data

**Table:**

C_10_H_8_BrN	*F*(000) = 440
*M**_r_* = 222.08	*D*_x_ = 1.636 Mg m^−^^3^
Monoclinic, *P*2_1_/*c*	Melting point: 343 K
Hall symbol: -P 2ybc	Mo *K*α radiation, λ = 0.71073 Å
*a* = 5.0440 (17) Å	Cell parameters from 1765 reflections
*b* = 13.467 (4) Å	θ = 2.2–26.0°
*c* = 13.391 (4) Å	µ = 4.50 mm^−^^1^
β = 97.678 (4)°	*T* = 291 K
*V* = 901.4 (5) Å^3^	Block, colourless
*Z* = 4	0.36 × 0.31 × 0.28 mm

### Data collection

**Table:**

Bruker SMART APEX CCD diffractometer	1765 independent reflections
Radiation source: fine-focus sealed tube	1039 reflections with *I* > 2σ(*I*)
graphite	*R*_int_ = 0.156
φ and ω scans	θ_max_ = 26.0°, θ_min_ = 2.2°
Absorption correction: multi-scan (*SADABS*; Sheldrick, 2004)	*h* = −6→6
*T*_min_ = 0.235, *T*_max_ = 0.286	*k* = −13→16
4668 measured reflections	*l* = −15→16

### Refinement

**Table:**

Refinement on *F*^2^	Primary atom site location: structure-invariant direct methods
Least-squares matrix: full	Secondary atom site location: difference Fourier map
*R*[*F*^2^ > 2σ(*F*^2^)] = 0.071	Hydrogen site location: inferred from neighbouring sites
*wR*(*F*^2^) = 0.195	H-atom parameters constrained
*S* = 1.01	*w* = 1/[σ^2^(*F*_o_^2^) + (0.0989*P*)^2^] where *P* = (*F*_o_^2^ + 2*F*_c_^2^)/3
1765 reflections	(Δ/σ)_max_ < 0.001
110 parameters	Δρ_max_ = 0.88 e Å^−^^3^
0 restraints	Δρ_min_ = −0.91 e Å^−^^3^

### Special details

**Table:**

Experimental. 1H NMR (CDCl_3_, 400 MHz) δ: 2.82 (s, 3H, CH_3_), 7.33(m, 2H, quinoline 3,6-H), 7.73 (dd, J=8.0 Hz, J=1.2 Hz, 1H, quinoline 7-H), 8.02 (m, 2H, quinoline 4,5-H).
Geometry. All e.s.d.'s (except the e.s.d. in the dihedral angle between two l.s. planes) are estimated using the full covariance matrix. The cell e.s.d.'s are taken into account individually in the estimation of e.s.d.'s in distances, angles and torsion angles; correlations between e.s.d.'s in cell parameters are only used when they are defined by crystal symmetry. An approximate (isotropic) treatment of cell e.s.d.'s is used for estimating e.s.d.'s involving l.s. planes.
Refinement. Refinement of *F*^2^ against ALL reflections. The weighted *R*-factor *wR* and goodness of fit *S* are based on *F*^2^, conventional *R*-factors *R* are based on *F*, with *F* set to zero for negative *F*^2^. The threshold expression of *F*^2^ > σ(*F*^2^) is used only for calculating *R*-factors(gt) *etc*. and is not relevant to the choice of reflections for refinement. *R*-factors based on *F*^2^ are statistically about twice as large as those based on *F*, and *R*- factors based on ALL data will be even larger.

### Fractional atomic coordinates and isotropic or equivalent isotropic displacement parameters (Å^2^)

**Table:**

	*x*	*y*	*z*	*U*_iso_*/*U*_eq_	
Br1	1.04425 (17)	0.41478 (5)	0.40291 (5)	0.0783 (4)	
N1	0.6713 (11)	0.2556 (3)	0.2991 (3)	0.0507 (12)	
C1	0.9974 (14)	0.3782 (4)	0.2655 (4)	0.0536 (16)	
C8	0.8122 (12)	0.3021 (4)	0.2325 (4)	0.0429 (13)	
C9	0.7845 (13)	0.2775 (4)	0.1287 (4)	0.0517 (15)	
C7	0.5039 (14)	0.1846 (4)	0.2646 (5)	0.0568 (16)	
C2	1.1397 (14)	0.4256 (4)	0.2022 (6)	0.0586 (16)	
H2	1.2578	0.4757	0.2266	0.070*	
C5	0.6014 (14)	0.2003 (5)	0.0957 (5)	0.0643 (18)	
H5	0.5778	0.1807	0.0285	0.077*	
C3	1.1122 (15)	0.4004 (5)	0.0989 (5)	0.0621 (18)	
H3	1.2156	0.4321	0.0561	0.074*	
C6	0.4624 (16)	0.1559 (5)	0.1622 (5)	0.070 (2)	
H6	0.3394	0.1064	0.1408	0.084*	
C4	0.9318 (15)	0.3287 (5)	0.0624 (5)	0.068 (2)	
H4	0.9066	0.3138	−0.0061	0.082*	
C10	0.3468 (14)	0.1364 (5)	0.3370 (6)	0.0694 (19)	
H10A	0.3969	0.0677	0.3444	0.104*	
H10B	0.1596	0.1412	0.3123	0.104*	
H10C	0.3821	0.1690	0.4011	0.104*	

### Atomic displacement parameters (Å^2^)

**Table:**

	*U*^11^	*U*^22^	*U*^33^	*U*^12^	*U*^13^	*U*^23^
Br1	0.1139 (9)	0.0735 (6)	0.0436 (4)	−0.0082 (4)	−0.0042 (4)	−0.0114 (3)
N1	0.059 (3)	0.050 (3)	0.042 (3)	0.011 (3)	0.004 (2)	0.005 (2)
C1	0.074 (5)	0.044 (3)	0.040 (3)	0.005 (3)	−0.001 (3)	−0.001 (2)
C8	0.037 (3)	0.050 (3)	0.040 (3)	0.012 (3)	0.001 (2)	−0.001 (2)
C9	0.054 (4)	0.063 (3)	0.037 (3)	0.009 (3)	0.002 (3)	−0.001 (3)
C7	0.059 (4)	0.053 (3)	0.059 (4)	0.011 (3)	0.009 (3)	0.007 (3)
C2	0.051 (4)	0.055 (3)	0.070 (4)	0.002 (3)	0.010 (3)	0.004 (3)
C5	0.057 (5)	0.083 (4)	0.050 (4)	0.003 (4)	−0.006 (3)	−0.015 (3)
C3	0.054 (5)	0.073 (4)	0.063 (4)	0.004 (4)	0.021 (3)	0.013 (3)
C6	0.080 (6)	0.063 (4)	0.062 (4)	−0.002 (4)	−0.008 (4)	−0.007 (3)
C4	0.081 (6)	0.085 (5)	0.039 (3)	0.013 (4)	0.012 (3)	0.004 (3)
C10	0.054 (4)	0.068 (4)	0.088 (5)	0.002 (4)	0.015 (4)	0.007 (4)

### Geometric parameters (Å, °)

**Table:**

Br1—C1	1.889 (6)	C2—H2	0.9300
N1—C7	1.318 (8)	C5—C6	1.344 (10)
N1—C8	1.365 (7)	C5—H5	0.9300
C1—C2	1.344 (9)	C3—C4	1.371 (10)
C1—C8	1.416 (8)	C3—H3	0.9300
C8—C9	1.418 (7)	C6—H6	0.9300
C9—C4	1.411 (9)	C4—H4	0.9300
C9—C5	1.422 (9)	C10—H10A	0.9600
C7—C6	1.414 (8)	C10—H10B	0.9600
C7—C10	1.481 (9)	C10—H10C	0.9600
C2—C3	1.412 (10)		
			
C7—N1—C8	118.0 (5)	C6—C5—H5	120.2
C2—C1—C8	122.1 (6)	C9—C5—H5	120.2
C2—C1—Br1	118.9 (5)	C4—C3—C2	119.5 (6)
C8—C1—Br1	119.0 (4)	C4—C3—H3	120.2
N1—C8—C1	120.5 (5)	C2—C3—H3	120.2
N1—C8—C9	122.8 (5)	C5—C6—C7	119.9 (7)
C1—C8—C9	116.7 (5)	C5—C6—H6	120.1
C4—C9—C8	120.8 (6)	C7—C6—H6	120.1
C4—C9—C5	122.4 (6)	C3—C4—C9	119.9 (6)
C8—C9—C5	116.8 (5)	C3—C4—H4	120.1
N1—C7—C6	122.9 (6)	C9—C4—H4	120.1
N1—C7—C10	117.6 (6)	C7—C10—H10A	109.5
C6—C7—C10	119.5 (7)	C7—C10—H10B	109.5
C1—C2—C3	120.9 (6)	H10A—C10—H10B	109.5
C1—C2—H2	119.6	C7—C10—H10C	109.5
C3—C2—H2	119.6	H10A—C10—H10C	109.5
C6—C5—C9	119.6 (6)	H10B—C10—H10C	109.5
